# Daily-Life Gait Quality as Predictor of Falls in Older People: A 1-Year Prospective Cohort Study

**DOI:** 10.1371/journal.pone.0158623

**Published:** 2016-07-07

**Authors:** Kimberley S. van Schooten, Mirjam Pijnappels, Sietse M. Rispens, Petra J. M. Elders, Paul Lips, Andreas Daffertshofer, Peter J. Beek, Jaap H. van Dieën

**Affiliations:** 1 MOVE Research Institute Amsterdam, Department of Human Movement Sciences, Vrije Universiteit Amsterdam, Amsterdam, the Netherlands; 2 EMGO+ Institute, Department of General Practice and Elderly Care, VU Medical Center, Amsterdam, the Netherlands; 3 MOVE Research Institute Amsterdam, Department of Internal Medicine, VU Medical Center, Amsterdam, the Netherlands; Ludwig-Maximilian University, GERMANY

## Abstract

Falls can have devastating consequences for older people. We determined the relationship between the likelihood of fall incidents and daily-life behavior. We used wearable sensors to assess habitual physical activity and daily-life gait quality (in terms of e.g. stability, variability, smoothness and symmetry), and determined their predictive ability for time-to-first-and-second-falls. 319 older people wore a trunk accelerometer (Dynaport MoveMonitor, McRoberts) during one week. Participants further completed questionnaires and performed grip strength and trail making tests to identify risk factors for falls. Their prospective fall incidence was followed up for six to twelve months. We determined interrelations between commonly used gait characteristics to gain insight in their interpretation and determined their association with time-to-falls. For all data -including questionnaires and tests- we determined the corresponding principal components and studied their predictive ability for falls. We showed that gait characteristics of walking speed, stride length, stride frequency, intensity, variability, smoothness, symmetry and complexity were often moderately to highly correlated (r > 0.4). We further showed that these characteristics were predictive of falls. Principal components dominated by history of falls, alcohol consumption, gait quality and muscle strength proved predictive for time-to-fall. The cross-validated prediction models had adequate to high accuracy (time dependent AUC of 0.66–0.72 for time-to-first-fall and 0.69–0.76 for -second-fall). Daily-life gait quality obtained from a single accelerometer on the trunk is predictive for falls. These findings confirm that ambulant measurements of daily behavior contribute substantially to the identification of elderly at (high) risk of falling.

## Introduction

Falls occur frequently among older people. The annual incidence of falls in people aged 65 and over is about one-third and about 15% of the people in this age group fall two or more times per year [1–3]. These falls are associated with high morbidity and mortality, making fall prediction and prevention important issues. Falls may be predicted by questionnaires and physical tests, and new insights suggest that wearable sensors may help to improve the accuracy of these predictions [4–7].

Recent technical developments in wearable sensors allow for ambulatory monitoring of human behaviour in daily life. Such methods can provide valid and reliable insight in an individual’s habitual daily activities, which seems particularly useful to numerate physical inactivity and to counter the associated detrimental effects on health our society is currently facing [8–10]. In the context of ageing, ambulatory monitoring of daily activity may be especially relevant for estimating fall risk. Physical inactivity is one of the main targets for fall prevention because of its detrimental effects on fitness, balance ability and fall risk (e.g. [11, 12]). Several studies showed that, in the long term, increasing habitual daily activity through training increases physical function and thereby protects against falls [11, 12]. However, some studies suggest that physical activity also increases exposure to risky situations and may hence increase the risk of falls in the short term; particularly in frail, fall-prone individuals due to incongruence of what they are able to do and actually do [5, 13, 14]. Objective assessment of habitual physical (in)activity may thus be valuable to identify those at risk of falls.

Besides the amount of daily activity, ambulatory measurements now also allow for quantification of quality of gait. The quality of daily-life gait seems to be a sensitive marker of intrinsic factors underlying balance ability as well as actual exposure to balance threats in daily life. Previous studies showed that daily-life gait quality characteristics derived from acceleration signals, such as walking speed and stride frequency, are related to fall risk [4–6, 15]. More sophisticated characteristics based on spectral ratios or temporal structure of trunk accelerations during gait may be more sensitive and provide insight in factors underlying the increased fall risk. Characteristics of daily-life gait such as the dominant frequency, root mean square, stride autocorrelation, spectral magnitude and width at stride frequency, percentage of spectral power under a predefined threshold, harmonic ratio, index of harmonicity, mean logarithmic rate of divergence and sample entropy have previously been associated with prospective falls [4–6]. The biological interpretation of such characteristics is still under debate (e.g. [16, 17]), and may be encumbered as they are based on similar methods and thus likely quantify similar aspects of gait. To improve our interpretation of these characteristics, we aim to establish their interrelations.

Recent studies revealed that prediction models comprising aspects of physical (in)activity and gait quality were able to predict falls with better accuracy than models based on questionnaires and physical tests [4–6]. Such prediction models showed similar predictive ability for falls, but differed considerable in the gait characteristics they comprised [4, 5]. A possible explanation may be the aforementioned similarities of these gait characteristics, or over fitting because of relative small sample sizes and lack of validation [18]. Our aim was therefore to decrease the risk of over fitting by dimension reduction prior to fitting a prediction model for falls. For generalization of these results and implementation of such models for risk detection, validation is of great importance. In addition, these previous studies employed logistic regression to predict binary *did* or *did not* fall. Survival models predicting the *time-to-*fall may be more sensitive and have the additional benefit that they allow for censoring as is often necessary in follow up studies. We therefore employed a survival model with cross-validation after dimensionality reduction to attain a generalizable fall prediction model.

## Methods

### Participants

This study was part of the FARAO project concerning fall-risk assessment in older people performed at the Vrije Universiteit Amsterdam. Participants were recruited between March 2011 and January 2014 in Amsterdam (the Netherlands) and surroundings via general practitioners, pharmacies, training groups, hospitals, and residential care facilities. Eligible persons were between 65 and 99 years of age, had a mini mental state examination score (MMSE [19]) of 19 to 30, and were able to walk at least 20 meters with aid of an assistive device if needed. All participants provided written informed consent and the protocol was approved by the medical ethical committee of the VU Medical Hospital (protocol 2010/290).

### Fall incidences

Prospective fall incidences were obtained through monthly telephone contact in addition to fall diaries to be filled out daily. When participants showed indications of impaired cognitive status (either an MMSE score below 24 or observed during questionnaire assessment), a caretaker was requested to assist with the fall diaries and accelerometer-wearing compliance. Follow-up was initially 6 months but was extended up to 12 months if the participant was willing to continue receiving monthly calls and keeping daily fall diaries and if the study duration allowed. Fall history was obtained by asking how frequent the participant had fallen in the 6 months and full year preceding the study.

### Accelerometry

Participants wore a tri-axial accelerometer (DynaPort MoveMonitor, McRoberts, The Hague, The Netherlands) for 8 consecutive days. This accelerometer had a sample rate of 100 samples/s and a range of -6g to +6g, and was worn dorsally on the trunk at the level of L5 using the supplied elastic belt. Participants were instructed to wear the accelerometer at all times, except during aquatic activities such as showering. The first and last 6 hours of the measurements were omitted from analysis to discard any possible artefacts caused by transportation to and from the participant's home.

#### Amount of physical activity

The amount of physical activity was quantified based on the accelerometer data. Bouts of non-wearing, locomotion, sitting, lying and standing were identified using the manufacturer’s algorithm (see supplementary material of Dijkstra et al. [8] for details). For days on which the accelerometer was worn more than 75% of the time [9], we calculated the total duration of locomotion, sitting, standing and lying, and the number of strides, average number of locomotion bouts, median and maximum duration of walking bouts, and the number of transitions to stance. These numbers were averaged over all eligible days to quantify habitual physical activity.

#### Gait quality characteristics

The daily-life gait quality was determined using methods described before [4, 5]. In brief, raw accelerations were aligned offline to anatomical axes based on the accelerometer's orientation with respect to gravity [20] and optimization of the left-right symmetry [21]. Subsequently all locomotion bouts exceeding 10s in duration were selected. These locomotion bouts were cut into 10-s windows for which we determined stride length from vertical trunk displacement assuming compass gait [22], stride frequency, and their product, walking speed [22]; gait intensity expressed as the root mean square of the signal; gait variability expressed as stride-to-stride variability in walking speed, stride frequency and stride length, the autocorrelation at the dominant period [23], the magnitude and width of the dominant period in the frequency domain [6, 24] and the percentage of power below 0.7 Hz [15]; gait symmetry expressed as the harmonic ratio [17, 25]; gait smoothness expressed as the index of harmonicity [26]; and gait complexity expressed as the mean logarithmic rate of divergence per stride using Wolf's method [27] and sample entropy [28]. Median values of characteristics over all 10-seconds windows were selected for further analysis to minimize problems of non-stationarity and to avoid bias by differences in data series length [29–31]. Where appropriate, characteristics were determined for each of the three directions of acceleration, i.e. anteroposterior (AP), mediolateral (ML) and vertical (VT).

### Questionnaires and tests of fall-risk factors

During a home visit at which participants started wearing the accelerometer, descriptive characteristics such as age, weight, height, living conditions and the use of a walking aid were obtained. Moreover, validated questionnaires and tests on fall-risk factors were completed. These comprised the LASA fall-risk profile [3], cognitive function (Mini Mental State Examination; MMSE [19]), executive functioning (trail making test parts A & B; TMT-A & TMT-B [32]), fear of falling (16-item fall efficacy scale; FES-I [33]) and depressive symptoms (30-item geriatric depression scale; GDS [34]). The LASA fall-risk profile includes questions concerning dizziness, independence in daily life, having pets, alcohol consumption, education duration and required the measurement of hand grip strength, which was quantified using a handgrip dynamometer (TKK 5401, Takei Scientific Instruments, Tokyo, Japan).

### Statistical analysis

Numerical variables were transformed to *z*-scores and dichotomous variables were transformed to -1 and +1 coding. We determined Pearson’s correlations between the 39 estimated gait quality characteristics (6 general characteristics and 11 characteristics in 3 directions). Correlations between 0.7 and 1.0 were considered strong, between 0.4 and 0.7 moderate and between 0 and 0.4 weak.

To determine univariate associations between each of the gait quality characteristics and time-to-first-fall and time-to-second-fall, we used an accelerated failure time (AFT) model. This survival model is analogous to a Cox proportional hazards model in that it allows for censoring as required given the different follow-up times in the current study, and has the benefit of the estimation of a baseline hazard, which is essential for prediction models. The underlying idea of this regression technique is that all cases will eventually experience the event and that certain factors will accelerate the time to this event. We used a shape of 1 and scale of 1 for the underlying generalized gamma distribution, effectively an exponential distribution, as this fitted the data best.

Prior to fitting multivariate models, we performed a principal component analysis (PCA) as data reduction technique. The number of principal components was determined using an eigenvalue of 1 as stopping criteria and results were rotated using a varimax rotation with Kaiser normalization. The resulting factors were coined according to the variables that weighted most heavily on each factor and subsequently used as input for multivariate AFT models with time-to-first-fall and time-to-second-fall as outcomes. Factor scores were added stepwise to the AFT model using forward selection until their addition did no longer contribute significantly (*p*>0.05). The outcome of the prediction models, i.e. hazard per month, was transformed to probability of survival. The prediction models were subsequently validated using leave-one-out validation and classification accuracy was determined with time-dependent receiver operator characteristics (ROC) curves with bootstrapping to obtain 95% confidence intervals.

## Results

**Fig 1:**
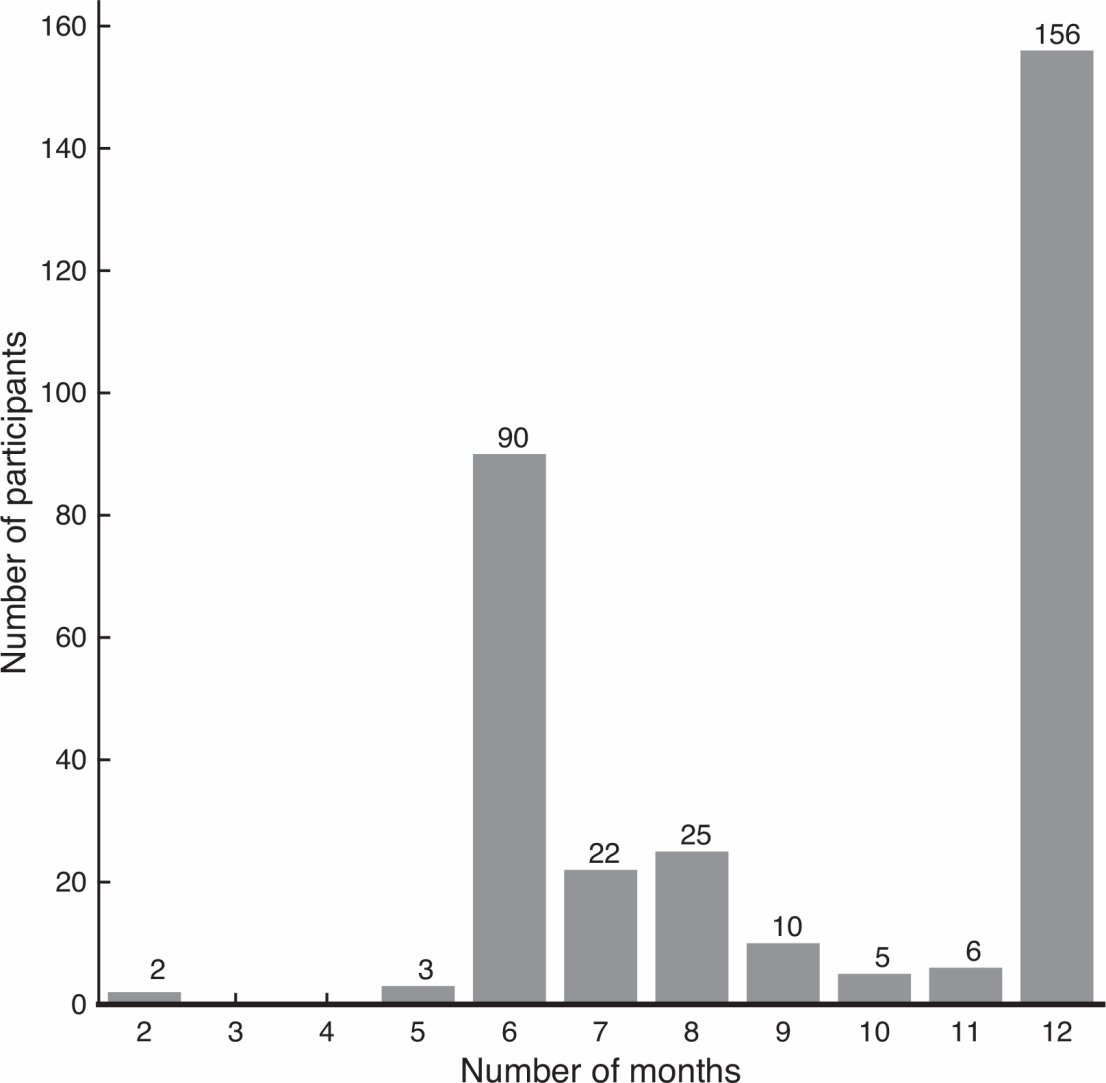
Fall follow-up durations. Drop out before 1 year resulted from serious injury or death (N = 5), opting not to continue after 6 months (N = 52) or the end of the study (N = 106).

A total of 319 older people were included in this study, out of which 51% was female and 90.3% was community-dwelling (Table 1). Participants were on average 75.5 (SD 6.9) years old, 170.6 (SD 8.8) cm tall and weighed 74.3 (SD 13.5) kg. They had a mean MMSE score of 27.6 (SD 2.3; 17 people had a score below 24, indicating mild cognitive impairment). 224 out of 276 people who were asked agreed to extend follow up after 6 months. The duration of the fall follow-up ranged between 2 and 12 months with a median duration of 11 months (IQR 6), see Fig 1.

**Table 1:** Baseline characteristics of the participants.

	All participants	Participants with ≥ 75% wear time and ≥ 50 locomotion episodes exceeding 10s
*N*	319	294
Age (years)	75.5 (6.9)	75.3 (6.8)
Male gender (%)	49.5%	49.2%
Length (m)	1.71 (0.09)	1.71 (0.09)
Weight (kg)	74.3 (13.5)	74.1 (13.4)
At least one fall in past year (%)	48.9%	48.8%
Two or more falls in past year (%)	24.8%	25.2%
Living independent (%)	90.3%	91.4%
Use of a walking aid (%)	18.5%	16.9%
Cognitive function (MMSE score)	27.6 (2.3)	27.7 (2.2)
Cognitive processing speed (time in s for TMT-A)	51.5 (25.0)	50.8 (23.6)
Executive functioning (time in s for TMT-B)	120.1 (56.7)	114.1 (55.0)
Hand grip strength (kg)	57.0 (19.9)	57.1 (19.9)
Depressive symptoms (GDS score)	4.8 (4.5)	4.8 (4.5)
Fear of falling (FESi score)	20.5 (5.6)	20.5 (5.6)
LASA fall risk profile (score)	5.34 (4.45)	5.19 (4.41)

Values represent mean (SD) or percentage.

The amount of physical activity of 21 participants was excluded because they had failed to comply with wearing the accelerometer at least 75% of any day [9]. Gait quality characteristics of 18 participants were excluded because they did not exhibit sufficient (> 50 in total) locomotion bouts exceeding 10 seconds [15]. One or more items in the questionnaires were missing for 12 participants, which were imputed by mean values. This left data of gait quality characteristics for 301 participants and complete data of 294 participants for further analysis. People excluded because of wear compliance or insufficient locomotion bouts were generally older (78.5 vs. 75.3 years; *p* = 0.025), were less often community dwelling (76.0 vs. 91.4%; *p* < 0.0001), and made more often use of a walking aid (40.0 vs. 16.9%; *p* = 0.012). See Table 1 for baseline characteristics.

### Correlations between gait quality measures

Correlations between gait quality characteristics estimated from different acceleration directions were frequently moderate to strong and often stronger between VT and AP directions than between other directions (see S1 Table). Exceptions were the index of harmonicity, amplitude of the dominant frequency and width of the dominant frequency. The index of harmonicity in VT exhibited a weak negative correlation with ML (*r* = -0.39) and AP (*r* = -0.18). The amplitude of the dominant frequency in VT exhibited a weak negative correlation with ML (*r* = -0.33) and a weak positive correlation with AP (*r* = -0.27); the amplitude of the dominant frequency in ML and AP did not correlate significantly (*p* = 0.80). The width of the dominant frequency in VT did not correlate significantly with ML (*p* = 0.35) and moderately with AP (*r* = 0.46); ML and AP correlated weak (*r* = 0.17).

Correlations between different gait quality characteristics were also often moderate to strong (see S1 Table). Walking speed was strongly correlated with step length, and with root mean square and range in VT and AP, as well as with amplitude of dominant frequency in VT and rate of divergence per stride in VT. Variability of stride time, stride length and stride speed were strongly correlated with each other and with autocorrelation in VT and AP direction, low frequency power in VT and AP, and harmonic ratio and rate of divergence per stride in VT. The index of harmonicity was strongly correlated with the amplitude of dominant frequency. Moreover, autocorrelation at the dominant frequency, harmonic ratio, amplitude of dominant frequency, and rate of divergence per stride were strongly correlated.

### Univariate associations with time-to-fall

Stride frequency, root mean square in VT and AP, autocorrelation in VT and AP, amplitude of the dominant frequency in VT and ML, width of the dominant frequency in AP, index of harmonicity in VT and ML, harmonic ratio in VT and AP, and rate of divergence per stride in VT and AP were all significantly associated with time-to-first- and time-to-second-fall (all *p*<0.05, see Table 2). Range in AP was only associated with time-to-first fall and not to time-to-second fall, although hazard ratios were comparable (0.82 (95% CI 0.68–0.99) vs. 0.84 (95% CI 0.64–1.10)). Walking speed, stride length, amplitude of the dominant frequency in AP and sample entropy in ML were only associated with time-to-second-fall (all *p*<0.05). None of the physical activity characteristics was associated with time-to-first- or time-to-second-fall (all *p*≥0.06).

**Table 2:** Univariate associations between gait quality and physical activity with time-to-fall.

	Mean (SD)	First fall (HR [95% CI])	Second fall (HR [95% CI])
**Walking speed**	0.83 (0.20)	0.84 [0.70–1.00]	**0.70 [0.53–0.91]**
**Stride frequency**	0.86 (0.08)	**0.83 [0.70–0.98]**	**0.75 [0.59–0.95]**
**Stride length**	1.07 (0.20)	0.89 [0.75–1.06]	**0.78 [0.61–1.00]**
**Root mean square VT**	1.75 (0.51)	**0.78 [0.65–0.95]**	**0.71 [0.53–0.95]**
Root mean square ML	1.21 (0.27)	0.93 [0.78–1.10]	1.04 [0.82–1.33]
**Root mean square AP**	1.26 (0.28)	**0.78 [0.65–0.93]**	**0.75 [0.58–0.98]**
Range VT	11.36 (2.88)	0.86 [0.72–1.03]	0.84 [0.64–1.10]
Range ML	8.79 (2.80)	0.92 [0.77–1.09]	1.01 [0.79–1.29]
**Range AP**	8.55 (2.43)	**0.82 [0.68–0.99]**	0.84 [0.64–1.09]
Walking speed variability	0.07 (0.02)	1.09 [0.92–1.29]	0.97 [0.76–1.24]
Stride time variability	7.53 (4.09)	1.08 [0.94–1.25]	1.12 [0.92–1.37]
Stride length variability	0.06 (0.02)	1.08 [0.91–1.27]	0.97 [0.76–1.24]
**Autocorrelation at dominant period VT**	0.45 (0.16)	**0.82 [0.69–0.97]**	**0.72 [0.57–0.92]**
Autocorrelation at dominant period ML	0.34 (0.11)	1.01 [0.85–1.20]	1.09 [0.86–1.38]
**Autocorrelation at dominant period AP**	0.40 (0.11)	**0.84 [0.70–1.00]**	**0.75 [0.58–0.97]**
**Magnitude of dominant period in frequency domain VT**	0.62 (0.21)	**0.83 [0.70–0.98]**	**0.69 [0.53–0.88]**
**Magnitude of dominant period in frequency domain ML**	0.37 (0.16)	**1.18 [1.01–1.38]**	**1.28 [1.03–1.59]**
**Magnitude of dominant period in frequency domain AP**	0.52 (0.12)	**0.94 [0.79–1.12]**	**0.75 [0.58–0.97]**
Width of dominant period in frequency domain VT	0.76 (0.20)	1.09 [0.95–1.25]	1.10 [0.92–1.32]
Width of dominant period in frequency domain ML	0.77 (0.08)	0.89 [0.74–1.08]	0.80 [0.59–1.09]
**Width of dominant period in frequency domain AP**	0.72 (0.06)	**1.24 [1.09–1.40]**	**1.30 [1.14–1.49]**
Percentage of power under 0.7 HZ VT	0.19 (0.19)	1.04 [0.92–1.18]	1.09 [0.93–1.28]
Percentage of power under 0.7 HZ ML	8.29 (6.86)	1.07 [0.92–1.24]	1.18 [0.97–1.43]
Percentage of power under 0.7 HZ AP	8.48 (5.96)	1.11 [0.97–1.28]	1.19 [0.99–1.43]
**Index of harmonicity VT**	0.70 (0.15)	**0.84 [0.72–0.98]**	**0.72 [0.59–0.88]**
**Index of harmonicity ML**	0.49 (0.22)	**1.21 [1.02–1.44]**	**1.35 [1.05–1.74]**
Index of harmonicity AP	0.72 (0.09)	1.16 [0.98–1.39]	1.09 [0.85–1.39]
**Harmonic ratio VT**	2.19 (0.59)	**0.81 [0.68–0.96]**	**0.69 [0.53–0.90]**
Harmonic ratio ML	1.85 (0.35)	0.97 [0.82–1.16]	0.97 [0.76–1.26]
Harmonic ratio AP	1.82 (0.42)	**0.76 [0.63–0.91]**	**0.64 [0.49–0.85]**
**Mean logarithmic rate of divergence VT**	1.49 (0.29)	**1.21 [1.01–1.44]**	**1.46 [1.12–1.90]**
Mean logarithmic rate of divergence ML	1.73 (0.20)	1.00 [0.84–1.19]	0.92 [0.72–1.16]
**Mean logarithmic rate of divergence AP**	1.65 (0.22)	**1.21 [1.01–1.45]**	**1.45 [1.10–1.91]**
**Mean logarithmic rate of divergence per stride VT**	1.76 (0.40)	**1.23 [1.04–1.45]**	**1.48 [1.16–1.88]**
Mean logarithmic rate of divergence per stride ML	2.04 (0.30)	1.10 [0.93–1.30]	1.10 [0.86–1.41]
**Mean logarithmic rate of divergence per stride AP**	1.94 (0.31)	**1.29 [1.09–1.54]**	**1.61 [1.24–2.08]**
Sample Entropy VT	0.25 (0.07)	1.04 [0.92–1.18]	1.00 [0.81–1.25]
**Sample Entropy ML**	0.35 (0.06)	0.88 [0.74–1.06]	**0.66 [0.50–0.89]**
Sample Entropy AP	0.27 (0.08)	1.07 [0.93–1.23]	1.01 [0.81–1.27]
Duration of locomotion	1.23 (0.55)	0.97 [0.81–1.15]	0.85 [0.66–1.10]
Number of strides	6407 (2971)	0.95 [0.79–1.13]	0.83 [0.64–1.07]
Number of locomotion bouts	405 (144)	1.05 [0.88–1.25]	0.95 [0.74–1.21]
Maximum duration of locomotion bouts	313 (239)	0.95 [0.79–1.14]	0.82 [0.60–1.10]
Maximum number of strides in one locomotion bout	565 (508)	0.94 [0.78–1.13]	0.80 [0.58–1.11]
Median duration of locomotion bouts	6 (1)	0.94 [0.79–1.12]	0.87 [0.67–1.12]
Median number of strides in one locomotion bout	7 (1)	0.89 [0.75–1.06]	0.82 [0.63–1.07]
Duration of lying	9.65 (2.52)	0.96 [0.81–1.14]	0.91 [0.71–1.18]
Duration of sitting	9.12 (2.38)	1.04 [0.87–1.24]	1.13 [0.88–1.46]
Duration of standing	2.62 (0.96)	1.12 [0.95–1.32]	0.99 [0.78–1.26]
Number of transfers	136 (58)	1.13 [0.96–1.32]	1.22 [0.99–1.50]
Duration of unclassified activities	0.36 (0.15)	1.00 [0.85–1.18]	1.01 [0.80–1.28]

Hazard ratios (HR) with 95% confidence intervals (95% CI). VT: vertical, ML: mediolateral, and AP: anteroposterior direction. Boldface indicates associations significant at *p*<0.05. All mean (SD) values are in Standard Units (m, sec, m/sec), except total durations of activities, which are in hours.

### PCA and multivariate associations with time-to-fall

**Fig 2:**
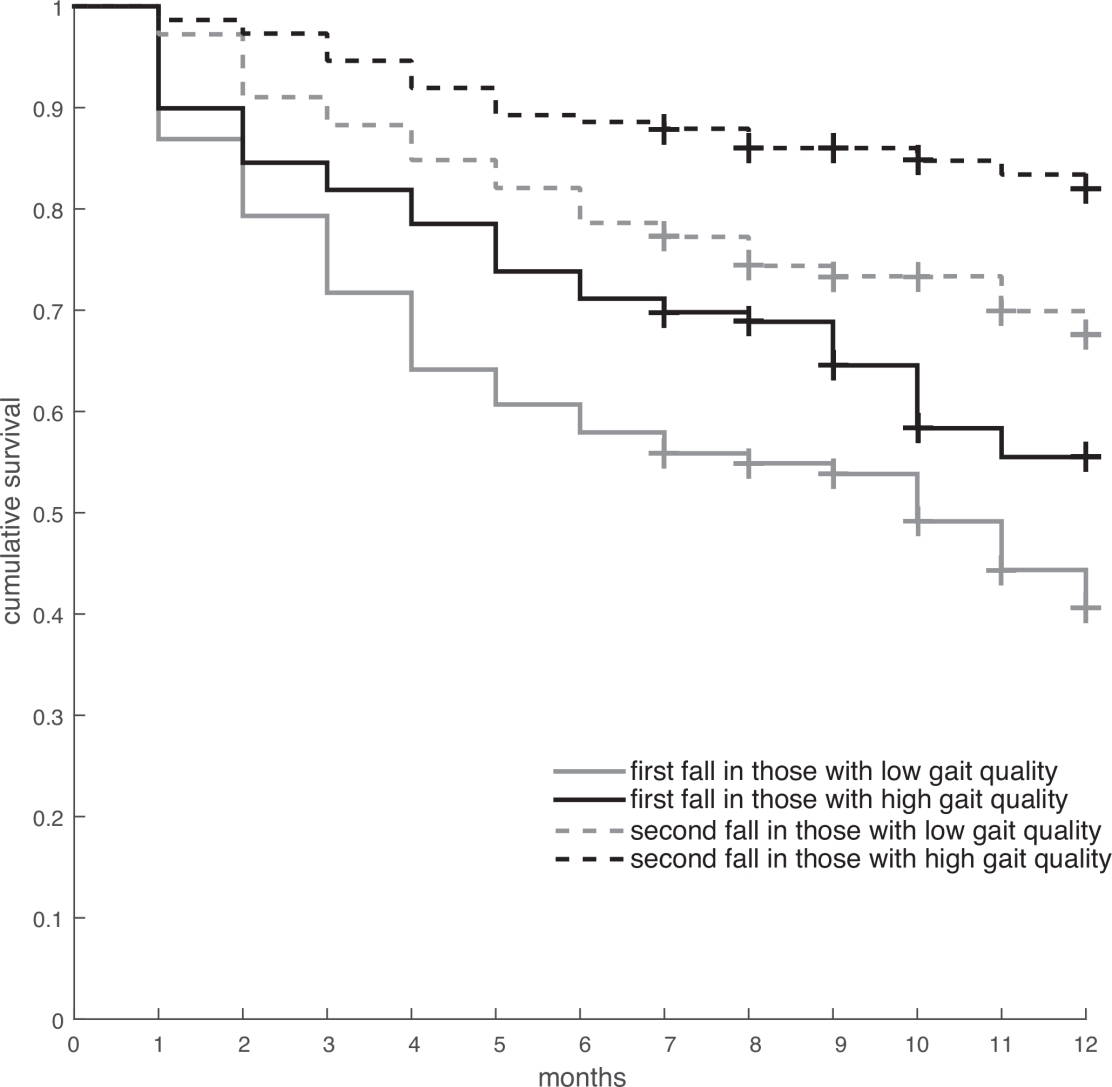
Kaplan-Meier curve depicting survival time for people with below and above average gait quality. + indicates censoring.

PCA revealed 18 principal components with an eigenvalue exceeding 1. Together, these principal components explained 80.5% of the variance in the questionnaire, tests, and accelerometry data (in total 75 variables). The varimax-rotated factor matrix can be found in S2 Table. The 18 factors reflected aspects which we coined gait quality, vigour, ML balance, physical activity, complexity, strength, disability, maximal gait duration, transfers, slow movements, history of falls, executive function, fear and depression, physical inactivity, cognition, body composition, alcohol consumption, and solace. The gait quality factor (which had a negative association with fall risk) was explained for 93% by autocorrelation at dominant period in VT (β = -0.72), root mean square of the signal in ML (β = 0.18), index of harmonicity in ML (β = 0.27) and magnitude at dominant period in frequency domain in AP (β = -0.20). The Kaplan Meier plot of survival times for people above and below average of this gait quality factor can be found in Fig 2.

The multivariate prediction model for time-to-first-fall comprised 3 factors: history of falls, alcohol consumption and the factor on which almost all gait quality variables loaded. This prediction model exhibited a cross-validated area under the curve between 0.66 and 0.72, which decreased slightly with follow-up (Table 3).

**Table 3:** Predictive ability of multivariate prediction model for time-to-first-fall.

Time-to-first-fall	Month											
	1	2	3	4	5	6	7	8	9	10	11	12
**Area under the ROC curve**	0.69	0.72	0.69	0.71	0.69	0.67	0.66	0.66	0.66	0.67	0.67	0.66
**Lower bound 95% confidence interval**	0.58	0.63	0.62	0.64	0.62	0.59	0.59	0.59	0.58	0.60	0.60	0.58
**Upper bound 95% confidence interval**	0.78	0.79	0.76	0.77	0.75	0.73	0.72	0.73	0.73	0.73	0.74	0.74
**Number of participants**	294	294	294	294	294	294	240	227	214	207	205	203
**Number of fallers**	34	53	68	84	96	104	109	111	116	125	131	134

The multivariate prediction model for time-to-second-fall included the same factors as the prediction model for time-to-first-fall with an additional factor related to strength. The final model included factors related to gait quality, fall history, strength and alcohol consumption. This prediction model had a cross-validated area under the curve between 0.69 and 0.76, which decreased slightly with follow-up (Table 4).

**Table 4:** Predictive ability of multivariate prediction model for time-to-second-fall.

Time-to-second-fall	Month											
	1	2	3	4	5	6	7	8	9	10	11	12
**Area under the ROC curve**	0.76	0.72	0.77	0.74	0.74	0.71	0.72	0.72	0.72	0.71	0.69	0.69
**Lower bound 95% confidence interval**	0.55	0.60	0.68	0.66	0.65	0.62	0.63	0.64	0.63	0.61	0.61	0.61
**Upper bound 95% confidence interval**	0.89	0.83	0.85	0.81	0.80	0.78	0.78	0.79	0.79	0.78	0.76	0.76
**Number of participants**	294	294	294	294	294	294	224	208	191	184	182	178
**Number of fallers**	6	17	25	34	42	48	51	56	57	58	62	65

## Discussion

We built upon previously reported fall prediction models that are based on the amount of daily activity and daily-life gait quality. We determined interrelations between gait characteristics to gain insight in their interpretation and subsequently assessed their predictive ability for time-to-fall. We further developed a prediction model for falls comprising daily-life activities, gait quality, and questionnaire data in a large and heterogeneous cohort of older people.

Gait quality characteristics were often highly correlated between methods even when they theoretically should describe different aspects. Walking speed was highly correlated with the root mean square and range in VT, which is not surprising since walking speed is estimated based on vertical displacement of the trunk. Walking speed was also correlated with the magnitude of the dominant period in the frequency domain and rate of divergence per stride in VT. A possible explanation could be that both are sensitive to walking speed, or that people with better capacities, and thus superior variability and complexity, simply walk faster. The index of harmonicity was highly correlated with the magnitude of the dominant period in the frequency domain, likely because the index of harmonicity is defined as the amplitude of this dominant frequency divided by itself plus the subsequent six harmonics [26]. Several variability measures were highly correlated with the rate of divergence per stride in VT. This may be due to the above-hypothesized relation with walking speed, but may also indicate that the rate of divergence per stride as estimated by Wolf’s method in daily life quantifies (aspects of) variability or its inverse, regularity. This notion is strengthened by its high correlation with the harmonic ratio and the autocorrelation at the dominant period. Future studies are required to determine whether gait quality characteristics in daily life indeed mainly quantify–aspects of–regularity of gait.

None of the physical activity characteristics was associated to time-to-fall. This is remarkable since we and others previously showed that the amount of gait and duration of lying were, independent or after controlling for gait quality, risk factors for falls [4–6, 14, 35]. A possible explanation could be that this relation is modified by physical capacity (such as gait quality) and that our group of participants was too heterogeneous to find such specific effects. Analysis of subgroups (e.g. [36]) may reveal such interactions in the future. We identified several gait quality characteristics that were associated with time-to-fall. These associations suggest that people with a higher risk for falls walked slower, less regular, less symmetric and less stable, more variable and less smooth in VT and AP, and less variable, smooth and predictable in ML, and these findings are comparable to those previously reported by us and others [4–6]. In line with these univariate findings, the negative loading of the gait quality factor after PCA suggests that people who display a lower autocorrelation at dominant frequency in VT (more variable), higher standard deviation of the signal in ML (more intense), higher index of harmonicity in ML (more smooth) and lower power at dominant frequency in AP (more variable) are at increased risk for falls.

Our results indicate that factors related to history of falls, alcohol consumption and gait quality predicted time-to-first-fall with an adequate to good accuracy (AUC 0.66–0.72). With the addition of the factor related to strength, these factors were able to predict time-to-second-fall with adequate to good accuracy (AUC 0.69–0.76). For prediction up to 6 months this accuracy is slightly better than that of commonly used prediction models for falls, which generally achieve AUCs ranging from 0.55 to 0.74 [37–39]. Predictive ability of both models slightly declined with time to follow-up. A possible explanation may be that gait characteristics’ predictive ability declines over time; however, future studies are required to verify this. The obtained prediction models performed slightly better in predicting second falls, which are less likely purely incidental and hence may be easier to predict.

The developed prediction models and gait quality component appear useful to identify those prone to falling, but may also be suitable to evaluate the effects of interventions. Future steps would be validation of here-developed method in an independent cohort and implementation in clinical setting. Intervention studies are required to determine whether these methods are sensitive enough to detect meaningful effects.

To the best of our knowledge, this study is the first to describe and take advantage of the correlations between gait quality measures to enhance fall prediction. However, it also has some limitations. We have used an extensive set of gait quality characteristics for the PCA, but adding other gait characteristics may affect the outcomes. We performed several sensitivity analyses in which we excluded variables to determine effects on the outcomes, and these appeared to be limited. We included questionnaire items with dichotomous variables in the PCA, which may not be desirable since it transforms the data such that the first principal component has the most variance and subsequent principal components have the highest variance possible while being orthogonal to the previous ones. To minimize the effect of these variables we coded them to be centered around zero with unit amplitude. Their number was low and excluding them from the analysis had limited effect on the final outcomes. Follow-up duration would have ideally been a full year (or longer) for all participants however organizational constraints prevented this. As result, the number of participants in the study decreased considerably after 6 months. The dropout was random for participant characteristics and the employed model was suitable for censoring, so we expect that this did not affect our results. We used a principal component analysis prior to fitting accelerated-failure time regression models; future studies could consider to employ feature selection and regularization methods such as LASSO or ridge regression. The population of this study, albeit large, is likely biased towards relatively healthy older people, since most were community-dwelling and their habitual walking speed was relatively high (i.e. 0.83 m/s). Additional analyses showed that predictive ability in individuals that used a walking aid in daily life, who are likely the more frail people, was comparable to that of the whole population (N = 49, 1^st^ fall AUC 0.59–0.75, 2^nd^ fall AUC 0.62–0.81). Studies in other populations are required to further establish the predictive ability of the developed methods for time-to-fall in more frail populations.

In conclusion, we showed that gait quality in daily life, expressed by separate characteristics or a composite factor based on these highly related variables, is predictive for both time-to-first and time-to-second falls in both univariate and multivariate models. The developed and cross-validated models for first and second falls can predict falls with adequate to good accuracy.

## Supporting Information

S1 Data Data underlying the finding described in the manuscript. (XLSX)

S1 Table Correlations between gait quality characteristics. Boldface indicates significance at *p*<0.05. Dark grey shade indicates correlations between different directions of acceleration within characteristics.
(DOCX)

S2 Table Loading of variables on Varimax-rotated principal components. Grey shade indicates loadings exceeding |0.3| which were used to coin factors.
(DOCX)
