# Supplementary material for: Daily-Life Gait Quality as Predictor of Falls in Older People: A 1-Year Prospective Cohort Study
Source: PLoS One. 2016 Jul 7;11(7):e0158623. doi: 10.1371/journal.pone.0158623 (PMC4936679; doi:10.1371/journal.pone.0158623)
Supplement: S1 Table — Boldface indicates significance at p<0.05. Dark grey shade indicates correlations between different directions of acceleration within characteristics. (DOCX) [file pone.0158623.s002.docx]

S1 Table: **Correlations between gait quality characteristics**

Boldface indicates significance at *p*<0.05. Dark grey shade indicates correlations between different directions of acceleration within characteristics.
