# Supplementary material for: Daily-Life Gait Quality as Predictor of Falls in Older People: A 1-Year Prospective Cohort Study
Source: PLoS One. 2016 Jul 7;11(7):e0158623. doi: 10.1371/journal.pone.0158623 (PMC4936679; doi:10.1371/journal.pone.0158623)
Supplement: S2 Table — Grey shade indicates loadings exceeding |0.3| which were used to coin factors. (DOCX) [file pone.0158623.s003.docx]

S2 Table: **Loading of variables on Varimax-rotated principal components**

|  | Factor | | | | | | | | | | | | | | | | | |
| --- | --- | --- | --- | --- | --- | --- | --- | --- | --- | --- | --- | --- | --- | --- | --- | --- | --- | --- |
|  | 1 | 2 | 3 | 4 | 5 | 6 | 7 | 8 | 9 | 10 | 11 | 12 | 13 | 14 | 15 | 16 | 17 | 18 |
|  | Gait quality | Vigour | ML balance | Physical activity | Complexity | Strength | Disability | Max gait duration | Transfers | Slow movements | History of falls | Executive function | Fear and depression | Physical inactivity | Cognition | Body composition | Alcohol consumption | Solace |
| Autocorrelation at dominant period VT | -.92 | .15 | -.14 | -.04 | -.14 | -.01 | -.05 | .02 | .02 | -.18 | -.04 | .00 | .02 | .00 | -.01 | .02 | .04 | -.03 |
| Mean logarithmic rate of divergence VT | .91 | -.15 | .12 | .06 | .04 | -.02 | .08 | -.13 | .03 | .10 | .03 | -.00 | -.04 | .02 | -.06 | .07 | .01 | .03 |
| Harmonic Ratio AP | -.87 | .05 | -.04 | .03 | -.13 | -.08 | .02 | .03 | -.06 | .01 | -.04 | .02 | -.03 | .01 | .04 | .02 | -.05 | -.01 |
| Autocorrelation at dominant period AP | -.86 | -.10 | -.18 | -.04 | .01 | -.12 | .07 | .19 | -.12 | .14 | -.03 | -.06 | .05 | .06 | .02 | -.11 | .04 | -.13 |
| Harmonic Ratio VT | -.85 | .11 | -.17 | .01 | -.10 | -.05 | -.04 | -.07 | .01 | -.15 | -.05 | .05 | -.03 | .02 | .02 | -.03 | -.03 | .06 |
| Mean logarithmic rate of divergence AP | .84 | .08 | .17 | .02 | .06 | .14 | -.07 | -.20 | .13 | -.14 | .01 | .05 | -.02 | -.07 | -.08 | .12 | .03 | .14 |
| Magnitude of dominant period in frequency domain VT | -.84 | .15 | .05 | .07 | -.12 | .01 | -.25 | .04 | -.00 | -.03 | -.00 | .02 | -.04 | -.05 | .09 | .17 | .00 | .05 |
| Mean logarithmic rate of divergence per stride VT | .83 | -.29 | .14 | -.05 | -.12 | .11 | .16 | -.12 | -.08 | .17 | .03 | -.00 | .01 | .06 | -.06 | -.13 | .01 | -.00 |
| Mean logarithmic rate of divergence per stride AP | .79 | -.16 | .18 | -.09 | -.15 | .31 | .06 | -.17 | -.04 | .00 | .03 | .03 | .03 | -.01 | -.08 | -.15 | .02 | .08 |
| Width of dominant period in frequency domain AP | .73 | -.02 | .08 | -.05 | .03 | -.07 | -.03 | .01 | -.13 | -.01 | .03 | -.02 | .03 | .08 | .10 | -.04 | -.00 | -.10 |
| Stride time variability | .71 | -.05 | .21 | .10 | .27 | .02 | -.08 | -.07 | -.03 | .52 | -.02 | .01 | -.08 | -.01 | .09 | .04 | .02 | .03 |
| Walking speed | -.65 | .50 | -.06 | .02 | -.01 | .15 | -.18 | .16 | .01 | -.18 | .08 | -.02 | -.15 | .05 | .04 | .07 | -.14 | .09 |
| Index of harmonicity ML | .62 | -.51 | -.27 | .00 | -.12 | .06 | .01 | -.01 | -.04 | -.00 | .08 | -.06 | .01 | .04 | .01 | .29 | -.07 | -.04 |
| Percentage of power under 0.7 Hz AP | .61 | -.27 | .05 | .09 | .30 | -.05 | -.03 | .01 | -.05 | .51 | -.02 | -.03 | .06 | .02 | .03 | .04 | .02 | -.02 |
| Stride length variability | .61 | .08 | .30 | .22 | .16 | .18 | -.19 | -.04 | .07 | .27 | .02 | -.03 | -.18 | .07 | .14 | .13 | -.15 | .21 |
| Index of harmonicity VT | -.60 | .15 | .17 | .15 | -.33 | .03 | -.34 | -.04 | .08 | .04 | .00 | .02 | -.12 | -.09 | .04 | .34 | .06 | .02 |
| Stride length | -.52 | .46 | .06 | .03 | -.23 | .36 | -.19 | .11 | -.02 | -.04 | .11 | -.04 | -.17 | .08 | .07 | -.02 | -.15 | .14 |
| Walking speed variability | .49 | .16 | .30 | .32 | .20 | .06 | -.21 | -.04 | .16 | .24 | .06 | .00 | -.20 | .04 | .14 | .23 | -.17 | .24 |
| Range AP | -.20 | .85 | .12 | .06 | -.13 | .02 | -.17 | .08 | -.02 | -.08 | .04 | -.01 | -.07 | .02 | .08 | .09 | .02 | .01 |
| Range VT | -.02 | .84 | .03 | .09 | -.03 | .04 | -.17 | .21 | -.03 | -.09 | .04 | -.02 | -.15 | .07 | .10 | .04 | -.10 | -.00 |
| Root mean square ML | -.05 | .81 | -.08 | .10 | -.26 | .04 | -.09 | -.09 | .23 | .01 | -.08 | -.04 | -.03 | -.09 | -.01 | -.15 | .17 | .08 |
| Range ML | -.09 | .78 | .15 | .04 | -.31 | .08 | -.06 | -.10 | .19 | -.05 | -.10 | -.03 | .02 | -.13 | -.03 | -.18 | .21 | .07 |
| Root mean square AP | -.39 | .78 | .08 | .12 | -.09 | -.05 | -.22 | .14 | -.05 | .05 | .04 | -.01 | -.08 | .07 | .13 | .01 | -.02 | .02 |
| Root mean square VT | -.42 | .74 | -.05 | .05 | .05 | .04 | -.20 | .25 | -.03 | -.11 | .03 | -.01 | -.12 | .05 | .12 | .05 | -.10 | -.00 |
| Index of harmonicity AP | .08 | -.71 | .04 | -.03 | -.39 | -.05 | .07 | -.16 | .07 | -.02 | -.03 | -.01 | .03 | -.04 | -.05 | -.28 | .00 | .08 |
| Magnitude of dominant period in frequency domain AP | -.56 | -.57 | -.01 | -.01 | -.28 | -.11 | .09 | .01 | -.09 | .13 | -.03 | -.07 | -.02 | .05 | .05 | -.28 | .01 | -.02 |
| Mean logarithmic rate of divergence ML | .28 | .01 | .87 | .01 | .01 | .16 | -.05 | -.10 | .10 | -.07 | -.05 | .04 | -.06 | -.02 | -.02 | .17 | .01 | .06 |
| Autocorrelation at dominant period ML | -.34 | -.11 | -.84 | -.09 | -.01 | -.13 | .10 | .02 | -.08 | .01 | .07 | -.05 | .06 | .02 | -.12 | .03 | .08 | -.10 |
| Harmonic ratio ML | -.27 | .01 | -.79 | .09 | -.09 | -.19 | .09 | -.09 | .03 | -.06 | -.02 | .01 | .01 | .04 | -.03 | -.15 | -.04 | .06 |

*Continued*

|  | Factor | | | | | | | | | | | | | | | | | |
| --- | --- | --- | --- | --- | --- | --- | --- | --- | --- | --- | --- | --- | --- | --- | --- | --- | --- | --- |
|  | 1 | 2 | 3 | 4 | 5 | 6 | 7 | 8 | 9 | 10 | 11 | 12 | 13 | 14 | 15 | 16 | 17 | 18 |
|  | Gait quality | Vigour | ML balance | Physical activity | Complexity | Strength | Disability | Max gait duration | Transfers | Slow movements | History of falls | Executive function | Fear and depression | Physical inactivity | Cognition | Body composition | Alcohol consumption | Solace |

| Mean logarithmic rate of divergence per stride ML | .33 | -.22 | .74 | -.14 | -.19 | .34 | .09 | -.10 | -.07 | .08 | -.02 | .01 | .02 | .02 | -.02 | -.13 | .01 | .01 |
| --- | --- | --- | --- | --- | --- | --- | --- | --- | --- | --- | --- | --- | --- | --- | --- | --- | --- | --- |
| Magnitude of dominant period in frequency domain ML | .36 | -.42 | -.70 | .06 | -.05 | -.09 | .00 | -.02 | -.04 | .06 | .12 | -.02 | -.05 | -.01 | .00 | .19 | -.10 | .02 |
| Width of dominant period in frequency domain ML | .17 | .06 | .68 | .07 | .09 | -.12 | -.01 | .09 | -.08 | .16 | -.10 | .01 | -.05 | .14 | .00 | -.09 | -.09 | -.10 |
| Number of locomotion bouts | -.08 | .11 | .07 | .87 | -.12 | -.10 | -.19 | .10 | .06 | .01 | -.02 | .01 | -.08 | .01 | .10 | -.03 | .01 | .06 |
| Duration of unclassified activities | .21 | .06 | -.01 | .77 | .01 | .01 | -.08 | .11 | -.01 | .08 | -.06 | .02 | .05 | -.07 | .00 | .13 | .15 | -.06 |
| Duration of standing | .06 | -.01 | -.12 | .76 | .02 | -.19 | -.06 | -.03 | -.33 | -.00 | .08 | -.01 | .02 | .07 | -.01 | -.02 | -.13 | .01 |
| Duration of locomotion | -.17 | .17 | .04 | .71 | -.01 | .05 | -.19 | .49 | .24 | -.02 | -.04 | .04 | -.08 | -.00 | .10 | -.07 | .04 | .03 |
| Number of strides | -.21 | .19 | .02 | .63 | .04 | -.04 | -.19 | .56 | .25 | -.05 | -.04 | .04 | -.09 | -.01 | .08 | -.02 | .03 | .05 |
| Sample entropy AP | .43 | -.04 | -.13 | -.07 | .80 | -.03 | -.01 | .02 | -.02 | -.02 | -.04 | -.02 | .02 | -.07 | -.04 | -.11 | .06 | .02 |
| Sample entropy ML | -.08 | -.13 | .24 | .08 | .78 | -.12 | -.12 | .17 | -.09 | .07 | .01 | -.01 | -.07 | .06 | .09 | .17 | -.12 | -.06 |
| Sample entropy VT | .29 | -.10 | .01 | -.05 | .74 | -.01 | -.01 | -.06 | -.03 | .36 | -.06 | .01 | .01 | -.10 | -.03 | .03 | .21 | -.05 |
| Width of dominant period in frequency domain VT | .49 | -.03 | .07 | -.04 | .70 | -.00 | .08 | .11 | -.16 | .05 | .00 | -.02 | -.02 | -.01 | .08 | -.12 | .00 | .03 |
| Height | .04 | .02 | .19 | -.08 | -.01 | .84 | -.03 | -.03 | .03 | .04 | .00 | -.07 | -.11 | .02 | .06 | -.05 | .03 | .07 |
| Female gender | .16 | .03 | .16 | -.10 | -.18 | .75 | .00 | .10 | .01 | -.00 | -.12 | -.04 | -.03 | -.02 | -.07 | .23 | .20 | -.05 |
| Handgrip strength | .10 | .17 | .22 | .04 | .03 | .75 | -.17 | .06 | .09 | .07 | -.21 | .00 | -.05 | -.06 | .10 | .11 | -.01 | -.01 |
| Weight | .11 | .05 | -.06 | -.11 | .05 | .73 | -.00 | -.19 | .24 | -.08 | -.02 | .00 | .03 | -.07 | -.06 | -.36 | .03 | .04 |
| Living independently | .05 | -.10 | .04 | -.02 | -.07 | -.03 | .81 | -.13 | -.02 | .01 | .02 | .01 | -.03 | .05 | -.03 | .09 | .00 | .03 |
| Inability to use public transportation | .02 | -.14 | -.06 | -.13 | -.01 | .02 | .76 | -.03 | -.02 | .08 | .03 | .02 | .18 | -.02 | -.10 | -.04 | -.05 | .13 |
| Use of a walking aid | .07 | -.27 | -.13 | -.16 | -.03 | -.02 | .62 | -.03 | -.00 | .03 | .04 | -.00 | .39 | -.08 | .01 | -.03 | -.02 | -.16 |
| Inability to descent and ascent a stair with 15 steps | .10 | -.22 | -.01 | -.22 | .01 | -.11 | .59 | -.03 | -.02 | -.02 | .15 | .00 | -.10 | -.08 | -.04 | -.26 | -.14 | -.06 |
| Inability to clip own toenails | .07 | -.19 | -.11 | -.08 | .09 | -.08 | .47 | -.08 | .04 | -.25 | -.01 | -.03 | .12 | -.09 | -.19 | -.01 | .14 | -.05 |
| Age | .03 | -.40 | -.04 | -.25 | -.13 | -.08 | .45 | .01 | -.08 | -.10 | -.05 | -.02 | .00 | -.01 | -.23 | .13 | .17 | -.19 |
| Maximum duration of locomotion bouts | -.25 | .17 | -.00 | .16 | .10 | .02 | -.09 | .86 | .06 | .01 | -.04 | .04 | -.02 | -.06 | .06 | .01 | -.00 | .04 |
| Maximum number of strides in one locomotion bout | -.21 | .18 | .00 | .16 | .09 | -.04 | -.10 | .86 | .06 | .02 | -.04 | .03 | -.03 | -.05 | .05 | .04 | -.02 | .06 |
| Median duration of locomotion bouts | -.05 | .13 | -.02 | .07 | -.04 | -.02 | -.04 | .05 | .87 | -.16 | .01 | -.01 | -.10 | .13 | .06 | .11 | -.06 | -.01 |
| Median number of strides in one locomotion bout | .10 | .07 | .01 | .02 | -.13 | .28 | -.01 | .11 | .80 | .00 | -.01 | -.04 | .02 | .16 | .02 | -.04 | -.01 | -.12 |

*Continued*

|  | Factor | | | | | | | | | | | | | | | | | |
| --- | --- | --- | --- | --- | --- | --- | --- | --- | --- | --- | --- | --- | --- | --- | --- | --- | --- | --- |
|  | 1 | 2 | 3 | 4 | 5 | 6 | 7 | 8 | 9 | 10 | 11 | 12 | 13 | 14 | 15 | 16 | 17 | 18 |
|  | Gait quality | Vigour | ML balance | Physical activity | Complexity | Strength | Disability | Max gait duration | Transfers | Slow movements | History of falls | Executive function | Fear and depression | Physical inactivity | Cognition | Body composition | Alcohol consumption | Solace |

| Number of transfers | -.01 | .11 | -.06 | .47 | .08 | -.05 | -.03 | -.06 | -.57 | -.08 | .09 | -.01 | -.04 | .11 | .02 | .10 | -.08 | -.06 |
| --- | --- | --- | --- | --- | --- | --- | --- | --- | --- | --- | --- | --- | --- | --- | --- | --- | --- | --- |
| Percentage of power under 0.7 Hz VT | .47 | -.14 | .13 | .00 | .24 | .02 | -.08 | -.01 | -.08 | .72 | -.06 | -.02 | .02 | -.03 | .01 | -.04 | .16 | -.06 |
| Percentage of power under 0.7 Hz ML | .49 | -.35 | -.04 | -.01 | .08 | .05 | .10 | .03 | -.21 | .62 | .00 | -.00 | -.00 | .08 | -.01 | -.09 | -.08 | -.05 |
| History of falls in past 6 months | .09 | .07 | -.03 | -.01 | -.03 | -.10 | .06 | -.02 | -.00 | -.01 | .87 | .08 | .11 | -.05 | .00 | .04 | .02 | -.00 |
| History of falls in past year | .06 | .02 | -.13 | -.01 | .01 | -.09 | .01 | -.06 | -.01 | -.05 | .87 | -.04 | .11 | -.06 | -.04 | -.04 | .03 | -.03 |
| Total LASA fall-risk profile score | .01 | -.13 | -.13 | .01 | -.08 | -.05 | .15 | .01 | -.09 | .01 | .55 | -.07 | .40 | .14 | .02 | .02 | .48 | .13 |
| Time on TMT B relative to time on TMT A | -.01 | -.02 | .04 | .02 | -.01 | -.05 | .00 | .04 | -.01 | -.01 | .01 | .99 | -.00 | -.01 | -.05 | -.00 | -.00 | -.02 |
| Time on TMT B | -.01 | -.02 | .04 | .02 | -.01 | -.05 | .01 | .04 | -.01 | -.01 | .01 | .99 | -.01 | -.00 | -.07 | .00 | -.00 | -.02 |
| Frequently experiencing dizziness | -.09 | -.02 | .00 | .11 | .04 | .00 | .17 | -.07 | .01 | .04 | .09 | .00 | .73 | .11 | .03 | .16 | -.26 | .10 |
| Fear of falling | .04 | -.22 | -.07 | -.12 | -.02 | -.18 | .07 | -.05 | -.00 | .05 | .21 | -.02 | .59 | -.01 | -.06 | -.19 | .09 | -.03 |
| Depression | .11 | -.19 | -.05 | -.15 | -.07 | -.05 | .08 | .02 | -.14 | -.23 | .21 | .00 | .49 | .03 | -.24 | -.08 | .10 | -.09 |
| Duration of sitting | -.02 | -.04 | -.01 | -.29 | .05 | .10 | .09 | -.02 | -.07 | -.01 | .03 | .01 | -.04 | -.90 | -.02 | .01 | -.04 | -.03 |
| Duration of lying | .04 | -.02 | .06 | -.28 | -.02 | .02 | -.01 | -.13 | .15 | -.00 | -.06 | .01 | .06 | .87 | .01 | .01 | .09 | .01 |
| Time on TMT A | .00 | -.20 | -.00 | -.11 | -.02 | .05 | .18 | -.04 | -.05 | .04 | .22 | -.06 | -.20 | .10 | -.70 | .06 | -.03 | -.00 |
| Followed higher education | .01 | -.01 | .02 | .04 | .01 | .17 | .01 | .08 | -.10 | .05 | .11 | -.08 | -.12 | .08 | .68 | .16 | .21 | .01 |
| Cognitive function | -.15 | .14 | .08 | .03 | .04 | -.08 | -.19 | .04 | .16 | .00 | .03 | -.16 | -.22 | .05 | .62 | -.10 | -.06 | .02 |
| Stride frequency | -.22 | .43 | -.11 | .21 | .29 | -.38 | -.19 | .03 | .24 | -.21 | -.01 | .02 | -.12 | -.05 | .01 | .45 | -.03 | .02 |
| Frequent alcohol consumption | .01 | .10 | -.03 | .06 | .10 | .21 | -.07 | -.02 | -.00 | .07 | .12 | .01 | -.12 | .12 | .17 | -.00 | .70 | .13 |
| Having a pet | .09 | .04 | -.04 | .02 | -.03 | -.01 | -.00 | .06 | -.09 | -.08 | .04 | -.01 | .04 | .04 | .04 | -.05 | .11 | .84 |
| Living alone | -.02 | .13 | .08 | -.04 | -.07 | .23 | -.01 | .12 | .05 | .31 | -.24 | -.07 | -.06 | -.04 | -.07 | .21 | .03 | .41 |

Grey shade indicates loadings exceeding |0.3| which were used t
